# New US Preventive Service Task Force recommendations for prostate cancer screening: a needed update, but not enough

**DOI:** 10.1590/S1679-45082017ED4151

**Published:** 2017

**Authors:** Marcelo Langer Wroclawski

**Affiliations:** 1 Hospital Israelita Albert Einstein, São Paulo, SP, Brazil.; 2 Faculdade de Medicina do ABC, Santo André, SP, Brazil.

Prostate cancer (PC) screening remains a controversial topic, although it has been studied for more than two decades. Since the 1990s, when the use of Prostate-Specific Antigen (PSA) began in clinical practice, the mortality rate from PC has decreased by around 50%. Prostate cancer screening policies, early diagnosis and treatment are pointed out as responsible to 45%-70% of this reduction.^(^ ^1^ ^)^ On the other hand, indiscriminate screening can lead to problems because of unnecessary prostate biopsies, and their possible side effects, such as infection and bleeding, and overdiagnosis and overtreatment of PC. These clinically non-significant tumors may be diagnosed and treated, perhaps, without any benefit to the patient. In addition, treatment sometimes causes potential side effects, such as urinary incontinence and erectile dysfunction –consequently worsening the quality of life.

The controversy became even greater when in 2012 the US Preventive Services Task Force (USPSTF) discouraged the use of PSA as a tool for PC screening (grade D recommendation), regardless of patient’s age.^(^ ^2^ ^)^

Of note is that USPSTF is an independent, volunteer panel of 16 American experts in prevention and evidence-based medicine. Currently, members of USPSTF are physicians (family practitioners, general practitioners, pediatricians, and gynecologists) and primary care nurses. However, interestingly, no urologist belongs to the panel.^(^ ^3^ ^)^

Considering the facts exposed above, undoubtedly, the main reason for screening and early diagnosis of any neoplasia is to reduce deaths from the disease. At the time that USPSTF recommended against the use of PSA, their arguments were based on two high-quality studies that assessed mortality in screened and nonscreened groups.

The first study included was the PLCO (Prostate, Lung, Colorectal, and Ovarian Cancer Screening Trial), a large randomized study conducted in 10 US centers that included 76,685 men between 1993 and 2001.^(^ ^4^ ^)^ Individuals were 55 to 74 years old, and were randomly assigned to receive or not receive annual evaluation with PSA for 6 years and digital rectal examination for 4 years, with minimal follow-up of 7 years. Biopsy was indicated if the PSA was higher than 4.0ng/mL or the result of digital rectal examination was abnormal. Detection of PC increased 22% in the Screened Group, but no significant difference was seen in cancer-specific mortality between groups. These results were not surprising because among other reasons, follow-up duration was very short (7 years) and, in the Control Group, there was contamination of the sample - more than 80% of individuals were evaluated by PSA at least once during the study.^(^ ^5^ ^)^ Therefore, the PLCO trial, truly compared two different screening forms and, therefore, the results must be interpreted carefully.

European researchers performed another important randomized trial about PC screening, the ERSPC (European Randomized study of Screening for Prostate Cancer).^(^ ^6^ ^)^ In the beginning of the 1990s, this trial sought to determine whether screening with PSA could reduce by 25% the risk for death from PC. The study included 182,160 men aged 55 to 69 years who were randomly assigned to undergo or not undergo screening every 2 to 4 years. Although, on average, each man in the intervention group had only 2.1 PSA assessments during the entire follow-up period, PSA levels that would lead to prostate biopsies were not uniform, ranging from 3.0 to 10.0ng/mL according to country. Because sextant biopsies were done (random, including six fragments), which currently are considered inadequate, the study showed a PC incidence of 8.2% in the Screened Group and 4.8% in the Control Group, with 20% relative reduction in the risk of death from this neoplasia, after a mean follow-up of 9 years. The Screened Group had a higher incidence of localized and low-risk disease, but the frequency of bone metastasis was reduced by 41%. The results showed the need to screen 1,410 men and treat 48 patients to prevent 1 death by PC. In an update of this study with a median follow-up of 11 years, results seemed more favorable, with reduction of 21% of risk for death from PC, the estimated number of needed to screen to prevent 1 death was 936, and the number needed to treat was 33.^(^ ^7^ ^)^

At that time, a third study had been published and, although it presented the best methodology, it had a smaller sample, and it was practically ignored by USPSTF. In the Göteborg study,^(^ ^8^ ^)^ 20,000 men aged 50 to 64 years were randomly assigned to biannual evaluation with PSA or a Control Group. At 14-year follow-up, the incidence of PC was 12.7% in the Screened Group and 8.2% in Control Group with 44% reduction in the risk of death from PC. It was estimated that 293 men would need to be screened and 12 would need to be treated to prevent 1 death by PC.

However, considering the findings of the PLCO and ERSPC, and the fact that 90% of tumors diagnosed were treated surgically or with radiotherapy,^(^ ^9^ ^)^ the USPSTF inferred that problems related to screening would be, in a last analysis, those related to biopsy needed for diagnosis and also problems associated with treatment. For every 1,000 men who were screened, 1 death from PC could be avoided at the expense of 30 to 40 men with urinary incontinence and erectile dysfunction, two severe cardiovascular events, and one deep venous thrombosis. Also, for every 3,000 screened men, 1 death from treatment complications may occur.^(^ ^10^ ^)^ For these reasons, in 2012, the report was issued against PC screening.

From that time on, a number of new data were published. The ERSPC was updated again, but now the mean follow-up was 13 years, and results reported were even more favorable. The estimated needed number to screen to prevent 1 death from PC was 781, and the number needed to treat was 27.^(^ ^11^ ^)^ This estimation would enable to avoid 3 cases of metastatic disease for each 1,000 men who are screened.^(^ ^12^ ^)^ In the Göteborg study, after a 18 years of follow-up, they observed the possibility to avoid one death for every 139 screenings and 13 diagnoses of PC.^(^ ^13^ ^)^

In addition, today, there is strong evidence that active surveillance in low-risk PC provides long-term cancer-specific survival similar to radiotherapy and surgery.^(^ ^14^ ^)^ Currently, this more conservative management has been applied in approximately one third of patients diagnosed with low-risk PC, therefore diminishing the complications of overtreatment.^(^ ^15^ ^)^

Recently, consequences related to USPSTF recommendations are being published. These studies report a significant reduction in screening with PSA among all ages. In addition to the reduction in biopsies requests and decrease of PC incidence, there is a trend to the diagnose of more advanced and high grade cases of PC. At the same time, diagnosis of metastatic disease has increased. These findings are worrisome because they avoid the early diagnoses and, when necessary, the adequate treatment mainly in young men with clinically significant and potentially fatal disease who could benefit from screening.^(^ ^16^ ^)^

This year, the USPSTF will update its recommendations for PC screening, and they have made available a draft for public comment.^(^ ^17^ ^)^ In this new version of recommendations the decision or not for screening must be individualized, and physicians are advised to recommend screening to their patients aged 55 to 69 years, considering the possible benefits and harms caused by the use of PSA for PC diagnosis. To the USPSTF, the screening provides the benefit of a small reduction in the chance of dying from PC, but many men exposed to PSA may experience damages related to screening such as false-positive results and some of them need additional tests and, sometimes, a prostatic biopsy, as well as overdiagnosis and overtreatment, which are associated with complications in this neoplasia treatment, such as urinary incontinence and erectile dysfunction (grade C recommendation).

Because there are no evidences to guide screening in men at high risk for PC, the USPSTF suggests the same recommendations of the general population, including black men and men with family history of cancer. The USPSTF continues to recommend that PC screening using PSA should be not adopted for men older than 70 years old (grade D recommendation).

Although a significant advance this new recommendation guideline can represent, they are still not ideal. Strategies for personalized screening and adapted for risk of each patient must be applied. Currently, more data has appeared to justify the assessment with PSA by around 40 years of age. In a 30 years follow-up, more than 90% of deaths because of PC occurred among men that, when they were aged 40 to 49 years, presented a PSA level greater than the median to their age. For this reason, those with PSA over 0.7ng/mL need, by 50 years of age, to adopt a rigorous screening strategy because they are in the higher risk group. On the other hand, for individuals with PSA levels lower than the median of their age, the screening protocol could be less frequent.^(^ ^18^ ^)^ When to stop screening is also controversial. Because patients are living longer it seems inappropriate to establish the 70 years as a cut-off age. The use of life expectancy as a parameter for screening older patients, perhaps, is the most adequate criteria.

New tools that seek to improve accuracy of prostatic biopsy have been developed and have gained more space in clinical practice. Tumor markers, such as 4k score and Prostate Health Index (PHI) can be used to improve the selection of patients who really need a biopsy. Multiparametric magnetic resonance imaging of the prostate can stratify the risk of clinically significant PC, and when this exam is associated with fusion guided biopsy, it diagnoses more precisely intermediate and high risk cases, which need active treatment.^(^ ^19^ ^)^ Data provided by these tests must be incorporated in screening protocols in order to reduce overdiagnosis, mainly in cases of non-clinically significant tumors.

To reduce overtreament, the diagnosis of PC must be separated from its active treatment. Surgery and/or radiotherapy should be offered for men with intermediate to high-risk tumors. In most of cases of low risk PC, the preferable option is active surveillance.^(^ ^20^ ^)^

The PC is the solid tumor with higher incidence among men, and the second leading cause of cancer death. Indiscriminate screening can cause problems, but non-screening certainly would cause decrease in survival rates. The new USPSTF recommendations suggest that shared decision should be made, after explanation of risks and benefits of screening, in patients aged 55 to 69 years old. Still, factors not approached in these recommendations such as race, family history, men younger than 55 years or older than 69 years need to be reassessed to, ideally, enable a proposal of an personalized scheme for PC screening based on risk of each man.
